# Novel *phlebovirus*-like-AYUT and *Stenotrophomonas maltophilia* bacterial co-infection in a *Rhipicephalus sanguineus* s.l. tick

**DOI:** 10.1007/s11259-021-09855-7

**Published:** 2021-11-02

**Authors:** Wachareeporn Trinachartvanit, Warissara Kaenkan, Wanwipa Nooma, Pattraporn Jeangkhwoa, Pakavadee Rakthong, Visut Baimai, Arunee Ahantarig

**Affiliations:** 1 Biodiversity Research Cluster, Department of Biology, Faculty of Science, Mahidol University, Rama 6 Road, 10400 Bangkok, Thailand; 2 Faculty of Science and Technology, Rajabhat Suratthani University, Mueang Surat Thani, Surat, 84100 Thani, Thailand; 3 Center of Excellence for Vectors and Vector-Borne Diseases, Faculty of Science, Mahidol University at Salaya, Phutthamonthon 4 Road, 73170 Nakhon Pathom, Thailand

**Keywords:** Tick-borne virus, *Stenotrophomonas maltophilia*, *Rhipicephalus sanguineus* s.l., *Stenotrophomonas maltophilia*

## Abstract

Tick-borne viruses and bacteria that can cause diseases of animals and humans have high impact and are of concern as significant threats to human health worldwide. In this research, we screened microorganisms related to those pathogens in ticks from dogs, a cat, and a cow. The techniques used were PCR, DNA sequencing and phylogenetic analysis to detect and classify the microorganisms [*Flavivirus*, severe fever with thrombocytopenia syndrome virus (SFTSV), *Phlebovirus*, *Coronavirus*, Canine Parvovirus, eubacteria, *Coxiella* and *Rickettsia*]. A novel virus named *Phlebovirus*-like-AYUT and *Stenotrophomonas maltophilia* bacteria were found in one individual tick (*Rhipicephalus sanguineus* s.l.) from a dog. All tick samples were negative for *Rickettsia*, while 9/21 (42.9 %) were positive for *Coxiella* bacteria. The novel virus “*Phlebovirus*-like-AYUT” (the name derives from Phra Nakhon Si Ayutthaya Province in Thailand) was resolved by phylogenetic analysis of the partial L segment by maximum likelihood (ML) method using MEGA X. The phylogenetic tree also indicated that the virus was related to *Phlebovirus* in brown dog ticks reported in Trinidad and Tobago. In contrast, *Phlebovirus*-like-AYUT was in a distinct clade from Lihan tick *Phlebovirus*-Thailand (LTPV), which was previously found in cow ticks, *Rhipicephalus microplus*, in Nan Province, Thailand. This study reports the *Stenotrophomonas maltophilia* bacterium with a novel *Phlebovirus*-like-AYUT in a brown dog tick. The roles of this bacterium in a virus-positive tick or in viral transmission from animal host requires further investigation.

## Introduction

The role of ticks in spreading pathogenic viruses has been recognized for more than 100 years. This includes the discovery of the *Flavivirus* Louping ill virus, which has been recognized as being responsible for severe encephalitis in sheep and other livestock (Stockman 1918). Tick-borne viruses (TBVs) that can pass on diseases to animals and humans have caused much concern because of the increasing prevalence of tick-borne viral diseases (TBVDs) and their important influence on human health (Shen et al. 2018). The *Phlebovirus* genus belongs to the *Phenuiviridae* family (Kuhn et al. 2021), including viruses transmitted mostly by phlebotomine sandflies and by mosquitoes and ticks (Elliott and Brennan 2014). Two novel tick-borne *Phleboviruses* (TBPVs) (1) severe fever with thrombocytopenia syndrome virus (SFTSV) and (2) Heartland virus (HRTV), appear to be related to serious human diseases and have resulted in deaths in eastern Asian countries and in the United States (Shen et al. 2018). *Phlebovirus* sequences (L-segment) have been repeatedly identified in both questing and feeding ticks, but particularly in *Rhipicephalus sanguineus* s.l. specimens (Pimentel et al. 2019).

In *Amblyomma cajennense* tick eggs in southeastern Brazil, *16 S* rRNA gene sequence examinations discovered 17 different types of bacteria, identified as *Serratia marcescens*, *Stenotrophomonas maltophilia*, *Pseudomonas fluorescens*, *Enterobacter* spp., *Micrococcus luteus*, *Ochrobactrum anthropi*, *Bacillus cereus* and *Staphylococcus* spp., separated into 12 phylogroups (Machado-Ferreira et al. 2015). *Stenotrophomonas maltophilia* is a gram-negative multidrug resistant organism (MDRO) that is most often related to respiratory infections in humans (Brooke 2012). In this study, a novel *Phlebovirus*-like-AYUT (newly named owing to its identification in a sample from Phra Nakhon Si Ayutthaya Province, Thailand) and *S. maltophilia* bacteria in the same individual dog tick (*R. sanguineus* s.l.) were detected.

## Materials and methods

### Tick collection and identification

Ticks were collected from four provinces in Thailand (Ranong, Phatthalung, Phra Nakhon Si Ayutthaya and Bangkok) in June 2021 and preserved in liquid nitrogen. The hosts of the collected ticks were dogs, a cat and a cow. The ticks were washed and identified to the species level using standard taxonomic keys (Tanskul and Inlao 1989) and underwent molecular identification with 16 S+1 and 16 S-1 primers (Black and Piesman 1994).

## Microorganism detection

DNA and RNA were extracted from the same individual tick using AllPrep DNA/RNA Mini Kit from Qiagen, Germany. In this work, *Rickettsia* and eubacteria (based on DNA) (Table 1), were investigated. Additionally, the primers PF1/PF2/PF3 (*Flavivirus*), SFTSV (SFTSV S segment), HRT-GL2759F/HRT-GL3276R (*Phlebovirus* L segment), CoVproF/CoVproR (*Coronavirus*) and CPV-2 F/CPV-2R (Canine Parvovirus) were used for RNA detection in the same set of DNA extraction samples (Table 1).

**Table 1:** List of primers used in this study

Microorganism	Primer	Gene product	Sequence (5’–3’)	Product size (bp)	Reference
*Flavivirus*	PF1	NS5	TGYRTBTAYAACATGATGGG	200	Cook et al. 2006
	PF2		GTGTCCCADCCDGCDGTRTC		
	PF3		ATHTGGTWYATGTGGYTDGG		
SFTSV	SFTS-1	N (S segment)	CAGCCAGTTTACCCGAACAT	560	Luo et al. 2015
	SFTS-2		GAAAGACGCAAAGGAGT		
	SFTS-3		TGGCTCCGCGCATCTTCACA		
	SFTS-4		AGAGTGGTCCAGGATTGCTGTGG		
*Phlebovirus*	HRT-GL2759F	RdRP (L segment)	CAGCATGGIGGIYTIAGRGAAATYTATGT	500	Matsuno et al. 2015
	HRT-GL3276R		GAWGTRWARTGCAGGATICCYTGCATCAT		
*Coronavirus*	CoVpro-F	3CLpro	KAAYGGBYTDTGGYTDG	Approximately 400	This study
	CoVpro-R		TCHADDTGRTGCATRTA		
Canine Parvovirus	CPV-2 Forward	VP2 protein	GTACATTTAAATATGCCAGA	452	Temuujin et al. 2019
	CPV-2 Reverse		ATTAATGTTCTATCCCATTG		
Eubacteria	fD1	*16 S* rRNA	AGAGTTTGATCCTGGCTCAG	1484	Weisburg et al. 1991
	rP2		ACGGCTACCTTGTTAGGACTT		
*Coxiella*	COX-16 S rRNA (FW)	*16 S* rRNA	GGGGAAGAAAGTCTCAAGGGTAA	520	Almeida et al. 2012
	COX-16 S rRNA (RW)		TGCATCGAATTAAACCACATGCT		
*Rickettsia*	Rr17.61p	17-kDa antigen	GCTCTTGCAACTTCTATGTT	434	Williams et al. 1992
	Rr17.492n		CATTGTTCGTCAGGTTGGCG		

## Phylogenetic analysis

The nucleotide sequences obtained from this study were aligned with other reference sequences available at GenBank using ClustalW in MEGA X. Suitable nucleotide substitution models were selected using MEGA X: Hasegawa-Kishino-Yano with gamma distribution (HKY+G) for *Phlebovirus* (L segment) and Kimura 2-parameter with gamma distribution (K2P+G) for *Stenotrophomonas*. The maximum likelihood (ML) method was used to infer phylogenetic trees using MEGA X with 1000 bootstrap replicates. Model with lowest Bayesian Information Criterion (BIC) score was selected. The nucleotide sequences in this study were submitted to GenBank.

## Results

### Tick identification

A total of 37 tick samples were identified as follows. There were (i) 11 *R. sanguineus* s.l. adult females and 9 males from 6 dogs in Ranong, Phatthalung, Phra Nakhon Si Ayutthaya provinces (6 ticks from Ranong and Phatthalung were pooled in 3 samples) (ii) 2 *R. microplus* adult females from a cow in Phatthalung province were pooled in 1 sample (iii) 11 *Rhipicephalus* nymphs from 2 dogs in Ranong and Bangkok (iv) 3 *Haemaphysalis* nymphs and 1 larva from a cat in Ranong [14 nymphal and larval ticks were pooled in 3 samples (within the same genus)] as shown in Table 2. Based on molecular identification using the 16 S mt rDNA gene, a representative example of *R. sanguineus* s.l. with accession number MW876246 from positive PCR results showed 100 % (429/429) identity with *R. sanguineus* s.l. (accession numbers MG651947, KC170744 and MT322611).

**Table 2:** Tick sample identification and microorganism detection (F = female, M = male, N = nymph, L = larva, P = pool, I = individual)

Location (Province)	Host	Tick species	No. of tick samples	No. of positive samples							
				*Flavivirus*	*Coronavirus*	Canine Parvovirus	*Phlebovirus*	SFTSV	Eubacteria	*Coxiella*	*Rickettsia*
Bangkok	dog (n = 1)	*Rhipicephalus* sp.	1P (8 N)	-	-	-	-	-	1 (1P)	-	-
Phra Nakhon Si Ayutthaya	dog (n = 3)	*R. sanguineus*	14I (7 F, 7 M)	-	-	-	1 (1 M)	-	11 (6 F, 5 M)	7 (4 F, 3 M)	-
Phatthalung	dog (n = 1)	*R. sanguineus*	1P (1 F, 1 M)	-	-	-	-	-	1 (1P)	1 (1P)	-
	cow (n = 1)	*R. microplus*	1P (2 F)	-	-	-	-	-	1 (1P)	-	-
Ranong	dog (n = 2)	*R. sanguineus*	2P (3 F, 1 M)	-	-	-	-	-	-	-	-
		*Rhipicephalus* sp.	1P (3 N)	-	-	-	-	-	1 (1P)	1 (1P)	-
	cat (n = 1)	*Haemaphysalis* sp.	1P (3 N, 1 L)	-	-	-	-	-	-	-	-
Total	Host = 9		21 samples (Total = 37 ticks; 14 N, 13 F, 9 M, 1 L)	0	0	0	1	0	15	9	0

### Microorganism detection

Using fD1-rP2 primers, we found positive PCR products for 71.4 % (15/21) of the samples. Nine of the 15 could be sequenced and 6 were too faint to be sequenced. Four samples were 95.3-99.7 % identical to *Moraxella osloensis* (accession number CP047226). Two eubacteria-positive bacteria showed 100 % identity with the *Coxiella*-like endosymbiont of *R. sanguineus* s.l. (1359/1359, 100 %; 719/719, 100 %) (accession number KU892220). One was close to *Hymenobacter gummosus* (1260/1319, 95.5 %) (accession number NR159132). The other one was close to the uncultured bacterium HJ-50 (99 %) (accession number KJ643976). The identification of *S. maltophilia* bacteria (accession number MZ348529) in an *R. sanguineus* s.l. tick from Phra Nakhon Si Ayutthaya Province that was 99.5 % (746/750) identical to *S. maltophilia* strain ES-5 bacteria (accession number MK537385), which may cause various serious infections in humans was found.

All tick samples were negative for *Flavivirus*, SFTSV, *Coronavirus* and Canine Parvovirus (Table 2). A male dog tick was positive for the *Phlebovirus* L segment (accession number MZ356165, the same individual tick as that positive for *S. maltophilia* bacteria). We named this virus *Phlebovirus*-like-AYUT in reference to its sampling location in Phra Nakhon Si Ayutthaya Province, Thailand.

All samples were negative for *Rickettsia* (17 kDa) bacteria, while 9/21 (42.9 %) were positive for the *Coxiella 16 S* rRNA gene [*Coxiella*-like endosymbiont of *R. sanguineus* s.l. KU892220 (100 %)]. Positive results of *Coxiella* bacteria were found in *Rhipicephalus* s.l. from the provinces of Ranong and Phra Nakhon Si Ayutthaya.

Several types of eubacteria were found in *R. sanguineus* s.l. in this study. Interestingly, co-infection of *Phlebovirus*-like-AYUT and *S. maltophilia* was found in an *R. sanguineus* s.l. tick from a dog from Phra Nakhon Si Ayutthaya.

#### Phylogenetic analysis of *Phlebovirus* and *Stenotrophomonas maltophilia* bacteria

**Fig. 1:**
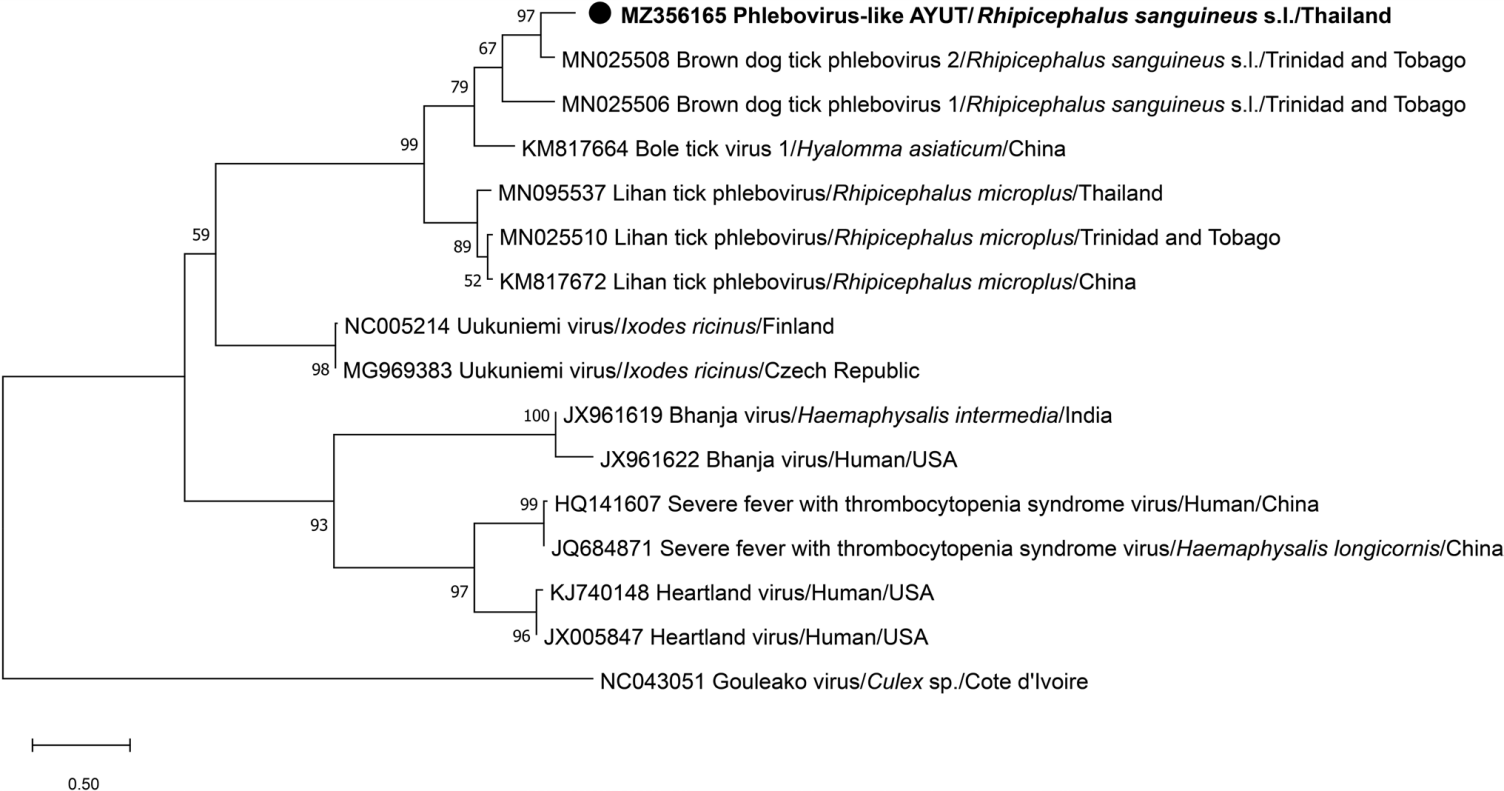
Phylogenetic relationship based on the partial sequence (687 bp) of *Phlebovirus* (L segment). The analysis was performed by the maximum likelihood method with the HKY+G model using 1,000 bootstrap replicates. Bootstrap values greater than 50 are shown above the nodes. Bold and circular sequences were obtained in this study. Gouleako virus was used as the outgroup

A BLASTn search showed that the partial L segment sequence from *R. sanguineus* s.l. in this study (*Phlebovirus*-like-AYUT) (accession number MZ356165) was related to brown dog tick *Phlebovirus* 2 (BDTPV2) (accession number MN025508) (82.6 %). It also shared 72.4 % and 70.3 % identity respectively with BDTPV1 (accession number MN025506) and Bole tick virus 1 (accession number KM817664). These two viruses have both been detected in *R. sanguineus* s.l. (from Trinidad and Tobago) and *Hyalomma asiaticum* (from China). The phylogenetic results based on the partial L segment revealed that the *Phlebovirus*-like virus identified in this study closely clustered with *Phlebovirus* from brown dog ticks from Trinidad and Tobago (accession number MN025508) (Fig. 1). In contrast, *Phlebovirus*-like-AYUT was in a distinct sister clade from Lihan tick *Phlebovirus*-Thailand (LTPV-Thailand), which has previously been found in *Rhipicephalus microplus*-infested cattle in Nan Province, Thailand. Furthermore, pairwise comparison showed that our sequence had 65.9 % similarity to the LTPV-Thailand virus sequence (accession number MN095537).

**Fig. 2:**
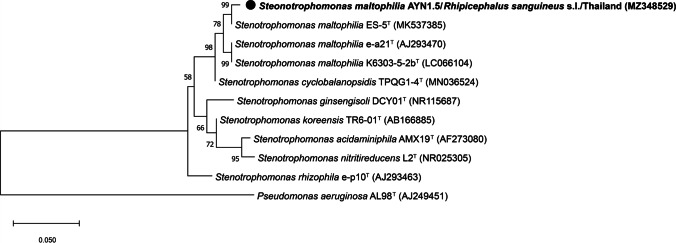
Phylogenetic relationship based on the partial sequence (450 bp) of the *16 S* rRNA gene of *Stenotrophomonas* species. The analysis was performed by the maximum likelihood method with the K2P+G model using 1,000 bootstrap replicates. Bootstrap values greater than 50 are shown above the nodes. The bold and circular sequences were obtained in this study. *Pseudomonas aeruginosa* was used as the outgroup

Phylogenetic results based on the partial sequence of the *16 S* rRNA gene revealed that *Stenotrophomonas* bacteria obtained in this study (accession number MZ348529) grouped with *S. maltophilia* strain ES-5 (accession number MK537385) (99.5 % identity by BLASTn) with strong support from a high bootstrap value (99 %) and was retrieved in the clade of *S. maltophilia*, which showed a clear separation from other groups (Fig. 2).

## Discussion

In 2015, LTPV was first found in *R. microplus* ticks from China and has since been found in other countries (Li et al. 2015; López et al. 2020). This virus is a *Phlebovirus*-like. Lihan tick *Phlebovirus*-Thailand was found in *R. microplus* from Thailand and was tentatively named LPTV-Thailand; it has 97 to 100 % amino acid identity with *Rhipicephalus*-associated *Phlebovirus* 1, a new strain of LTPV identified in *R. microplus* ticks from China (Temmam et al. 2019). In this work, an L RNA segment of virus in a brown dog tick sample from Phra Nakhon Si Ayutthaya Province was related to *Phlebovirus* in brown dog ticks from Trinidad and Tobago (82.6 % similarity) (Sameroff et al. 2019) and was clustered in the same clade with Bole tick virus and LTPV. This group shared a common ancestor with Uukuniemi virus. We named this virus *Phlebovirus*-like-AYUT and it is novel as indicated by percent DNA sequence similarity as compared with previously described forms.

In addition, *S. maltophilia* bacteria, which may cause serious infections in humans and have been isolated in several environments, was discovered here for the first time in an *R. sanguineus* s.l. tick from a domestic dog in Thailand. *Rhipicephalus sanguineus* s.l. usually infests dogs or domestic animals that are close to humans. This indicates the possibility that *R. sanguineus* s.l. may act as a vector to transmit disease from animals to humans. Such ticks that accidentally come into close contact with humans are very troubling because they may cause bacterial infection in humans. In Thailand, there have been no previous reports about this bacterium in *R. sanguineus* s.l. However, there are reports of the presence of *Stenotrophomonas* sp. in *Rhipicephalus* eggs and *R. microplus-*infested cattle from Colombia (Machado-Ferreira et al. 2015; Segura et al. 2020). *Stenotrophomonas maltophilia* has also been extracted from the eggs of *A. cajennese* tick (Machado-Ferreira et al. 2015). However, our study is the first report of *S. maltophilia* in *R. sanguineus* s.l. ticks from Thailand.

Diverse bacterial pathogens and soil organisms have previously been identified in ticks, e.g., *Pseudomonas*, *Acinetobacter*, uncultured gamma proteobacterium, *Stenotrophomonas* and *Enterobacter* spp. (Moreno et al. 2006). In the current study, several types of eubacteria (*Moraxella osloensis*, *Coxiella*-like endosymbiont, *Hymenobacter gummosus, S. maltophilia* and uncultured bacterium) were discovered in *R. sanguineus* s.l., in which *M. osloensis* and *S. maltophilia* are causative organisms of infections in humans. *Hymenobacter gummosus* is an environmental bacterium that has been isolated from water samples (Chen et al. 2017). *Moraxella osloensis* is a causative agent that can be found in several environments. A previous study reported that *I. scapularis* could be infected with *Moraxella* species (Moreno et al. 2006), while *S. maltophilia* bacteria are pathogenic in humans. Additionally, *Coxiella*-like endosymbionts have been detected in a wide variety of ticks, including *R. sanguineus* s.l. (Oskam et al. 2017).

Co-infection with *Phlebovirus*-like-AYUT and *S. maltophilia* was found in an *R. sanguineus* s.l. tick from a domestic dog in Phra Nakhon Si Ayutthaya Province for the first time. The co-infection of *Phlebovirus*-like-AYUT (RNA) and *S. maltophilia* bacteria (DNA) is an important issue because both are pathogenic to humans. The dog infected with these two pathogens exhibited weakness prior to mortality; however, the exact reasons for its death are not fully understood. The roles of this bacterium in a virus-positive tick or in viral transmission from animal hosts also requires further investigation.

## Data Availability

All data included in this study are available on request to the corresponding author.
